# 946. Implementation of a Burn Activation Protocol to Reduce Delays in Resuscitation

**DOI:** 10.1093/jbcr/irag033.513

**Published:** 2026-04-06

**Authors:** Lindsay DeSantis, Laurin Proctor, Samantha Allbritton, Lily Daniali, Ryan Endress, Wojciech Przylecki, Benson Pulikkottil

**Affiliations:** HCA HealthONE Swedish, Englewood, CO; HCA HealthONE Swedish, Englewood, CO; HCA HealthONE Swedish, Englewood, CO; HCA HealthONE Swedish, Englewood, CO; HCA HealthONE Swedish, Englewood, CO; HCA HealthONE Swedish, Englewood, CO; HCA HealthONE Swedish, Englewood, CO

## Abstract

**Introduction:**

Early resuscitation is critical in burn care, particularly within the first hour of presentation. At a Level I trauma center with an American Burn Association (ABA)-verified Burn and Reconstructive Center, gaps were identified in emergency department (ED) activations. Team members were notified individually rather than through a coordinated system, resulting in inconsistent team presence and delays in resuscitation. This study describes the development and implementation of a standardized Burn Activation protocol to improve communication, expedite team assembly, and ensure timely resuscitation.

**Methods:**

A multidisciplinary group reviewed workflows and identified barriers to rapid burn response. A protocol was created with defined clinical triggers for Burn Activation and a standardized notification system to alert all essential team members simultaneously. Training sessions for ED staff, Burn Intensive Care Unit (BICU) nurses, and ancillary services emphasized early fluid resuscitation and coordinated response. Key performance measures included time to resuscitation initiation and ED-to-BICU transport intervals.

**Results:**

The Burn Activation protocol standardized team notification and practice. Burn specialists were consistently present on ED arrival of a critical burn patient. Time from arrival to resuscitation initiation and ED-to-BICU transport improved, consistently occurring within one hour. Staff surveys reflected improved role clarity, streamlined workflow, and greater confidence in managing acute burn patients upon presentation to the ED.

**Conclusions:**

Implementation of a Burn Activation protocol at a Level I trauma and ABA verified burn center improved communication, standardized team notification, and decreased time to resuscitation. Structured activation criteria and coordinated processes enhanced efficiency, optimized early burn care, and ensured critically injured patients received timely, comprehensive treatment.

**Applicability of Research to Practice:**

This protocol offers a structured approach to standardize initial critical care evaluation of a burn injured patient in the ED and can serve as a model for other high-acuity care settings.

**Funding for the study:**

N/A.

